# Impact Performance Comparison of Advanced Bicycle Helmets with Dedicated Rotation-Damping Systems

**DOI:** 10.1007/s10439-019-02328-8

**Published:** 2019-07-24

**Authors:** Michael Bottlang, Alexandra Rouhier, Stanley Tsai, Jordan Gregoire, Steven M. Madey

**Affiliations:** Biomechanics Laboratory, Legacy Research Institute, Portland, OR 97232 USA

**Keywords:** Bicycle helmet, Rotation-damping system, Brain injury, Concussion, Oblique impact, Impact testing, Rotational acceleration, Slip liner

## Abstract

Bicycle helmets effectively mitigate skull fractures, but there is increasing concern on their effectiveness in mitigating traumatic brain injury (TBI) caused by rotational head acceleration. Bicycle falls typically involve oblique impacts that induce rotational head acceleration. Recently, bicycle helmet with dedicated rotation-damping systems have been introduced to mitigate rotational head acceleration. This study investigated the impact performance of four helmets with different rotation-damping systems in comparison to a standard bicycle helmet without a rotation-damping system. Impact performance was tested under oblique impact conditions by vertical drops of a helmeted headform onto an oblique anvil at 6.2 m/s impact speed. Helmet performance was quantified in terms of headform kinematics, corresponding TBI risk, and resulting brain strain. Of the four rotation-damping systems, two systems significantly reduced rotational head acceleration, TBI risk, and brain strain compared to the standard bicycle helmet. One system had no significant effect on impact performance compared to control helmets, and one system significantly increase linear and rotational head acceleration by 62 and 61%, respectively. In conclusion, results revealed significant differences in the effectiveness between rotation-damping systems, whereby some rotation-damping systems significantly reduced rotational head acceleration and associated TBI risk.

## Introduction

Bicycle riding provides clear health benefits.^26^,^56^ In the United States, the number of bicycle commuters increased by 61% between 2000 and 2012^37^ and an estimated 33 million children ride bicycles.^43^ However, bicycling is also the leading cause of sports-related head injuries treated in U.S. emergency rooms. Head injury from bicycle accidents caused 80,000 emergency department visits in 2015, with 13,000 of these visits including diagnosis of concussion and traumatic brain injury (TBI).^15^ The associated direct medical treatment cost exceeded $2 billion annually, not including the far greater costs due to work loss and quality-of-life loss.^22^

To date, bicycle helmets are the primary and most effective strategy to prevent TBI.^29^ Helmets are highly effective in reducing the risk of skull fracture, penetrating injury, and severe brain injury.^16^,^20^,^36^ Epidemiological studies have demonstrated that wearing a bicycle helmet reduces the head injury risk by up to 69%.^2^,^18^,^19^,^35^ Traditional bicycle helmets employ a rigid shell of expanded polystyrene foam (EPS) that dampens the impact, reduces the impact force, and in turn reduces head accelerations. Specifically, traditional helmets have been optimized to reduce linear acceleration of the head, as outlined in the mandatory impact test standard by the US Consumer Product Safety Commission (CPSC). This CPSC standard requires that a normal impact from a vertical drop of a helmeted headform onto a horizontal anvil results in less than 300 g linear acceleration of a test headform.^14^ However, in contrast to radial CPSC impacts, real-world impacts typically occur at impact angles of 30°–60°.^9^,^10^,^42^ Such oblique impacts induce both radial and tangential forces to the head, leading to both linear and rotational head acceleration.^36^,^57^ A large body of research has shown that concussions can readily be caused by head rotational kinematics, as measured by rotational acceleration or rotational velocity, which subjects brain tissue to shear forces that can induce diffuse axonal injury.^23^,^25^,^28^,^30^,^31^,^41^,^44^,^47^ Since axonal shear strain caused by rotational head acceleration is a predominant mechanism of brain injury,^38^ advanced helmet designs should specifically target mitigation of rotational kinematics, and should be tested in real-world oblique impacts.^49^

Recently, several bicycle helmet designs have been introduced that have dedicated rotation-damping systems for mitigation of rotational head acceleration to provide improved protection from brain injury.^1^,^7^,^20^,^27^ The most widely adopted strategy consists of a slip liner inside the helmet, termed Multidirectional Impact Protection System (MIPS AB, Täby, Sweden), that seeks to reduce rotational kinematics of the head by permitting sliding between the helmet and head during impact. More elaborate systems include helmet designs with double shells that are coupled by elastic dampers. This considerable variety in designs of rotation-damping systems suggests performance differences between these advanced helmet models, but there is a general lack of research data on the performance of advanced helmets with rotation-damping systems. Moreover, a recent study that tested two helmets with slip liners and eight traditional helmets without rotation-damping systems did not find that slip liners provided superior mitigation of rotational head acceleration compared to standard EPS helmets.^7^

The performance of rotation-damping systems cannot be evaluated with the standard CPSC impact-attenuation test for helmets, since it neither induces nor assesses rotational head acceleration. Therefore, an advanced helmet impact test method is required that simulates real-world oblique impacts, that allows for headform rotation upon impact, and that captures the rotational kinematics of the headform. A wide range of oblique impact test methods have been developed, including impact testing by guided free-fall onto an angled anvil,^7^,^21^,^27^,^33^,^40^ vertical drops onto a laterally translating impact surface^1^,^36^,^39^ and pendulum impact tests.^4^,^45^ Specifically for evaluation of advanced helmets with rotation-damping systems, the Legacy Biomechanics Laboratory has established the helmet impact testing (HIT) facility that allows for helmet testing under oblique impacts to measure rotational headform kinematics and to estimate the associated concussion risk. This study investigated the performance of four helmet designs with different rotation-damping systems in direct comparison to a standard bicycle helmet without a dedicated rotation-damping system. Oblique impact testing was conducted to capture headform kinematics, to estimate the corresponding concussion risk, and to compute strain in brain tissue. Results were used to test the hypothesis that performance differences between rotation-damping systems exist, and can be quantified in terms of mitigation of rotational head acceleration, associated concussion risk, and brain strain.

## Methods

### Helmets

**Figure 1:**
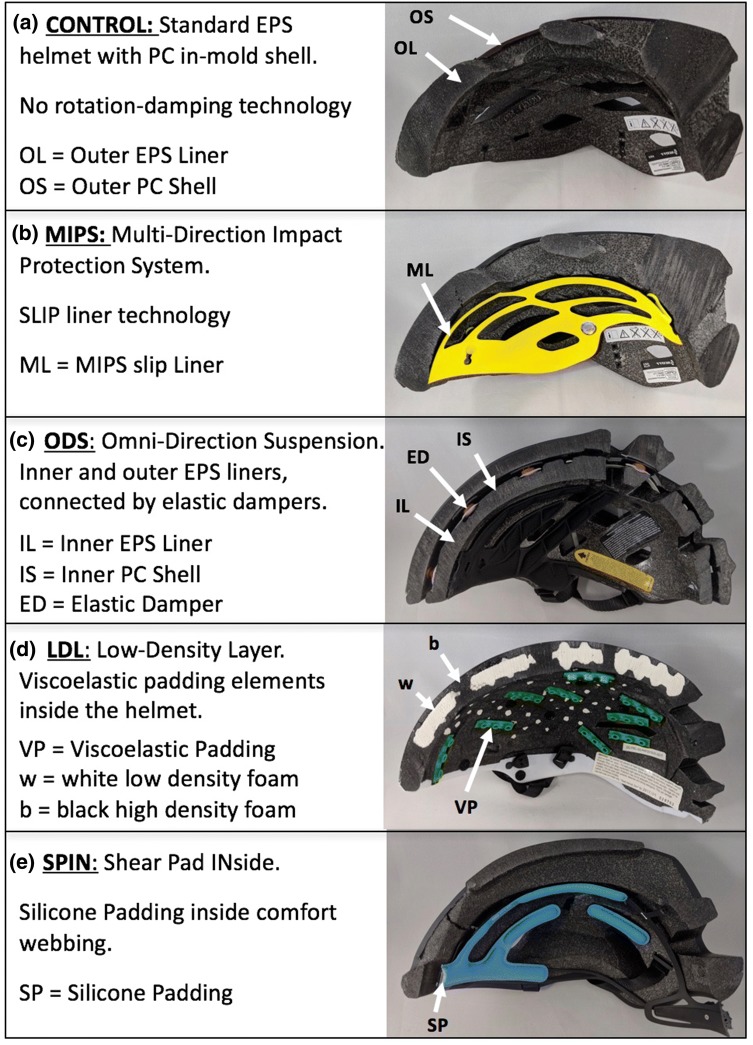
Mid-sagittal cross-section of (a) a standard helmet without a rotation-damping system, and (b–e) four helmet designs with different rotation-damping systems.

The test matrix included five bicycle helmets: Scott “ARX”; Scott “ARX +”; 6dhelmets “ATB-1T EVO”; Kali Protectives “Tava”; and the POC “Auric SPIN”. The Scott “ARX” (www.scott-sports.com, $100 list price) is a standard bicycle helmet without a rotation-damping system, and was selected for the CONTROL group. This midrange helmet had an in-molded polycarbonate micro-shell and a standard expanded polystyrene (EPS) liner (Fig. 1a). The same helmet design with a MIPS slip liner (Scott ARX Plus, www.scott-sports.com, $110 list price) was selected for the MIPS group. This Scott ARX Plus was the highest-scoring helmet of Consumer Reports’ 2016 Bike Helmet Ratings.^13^ The MIPS low friction liner is elastically suspended to allow for 10–15 mm motion between the helmet and head (Fig. 1b). The “ATB-1T EVO“helmets (www.6dhelmets.com, $210 list price) had dual EPS liners, connected by an array of elastomeric dampers that formed an Omni-Directional Suspension (ODS) in the ODS group (Fig. 1c). “TAVA” helmets (www.bikes.kaliprotectives.com, $240 list price) had a low density layer (LDL) technology, consisting of viscoelastic padding elements inside the helmet to reduce rotational impact forces in the LDL group (Fig. 1d). “TAVA” helmets also had a dual density EPS liner. “AURIC SPIN” helmets (www.pocsports.com, $220 list price) contained SPIN (Shear Pad Inside) technology that embeds silicone padding inside the comfort webbing of the helmet fit system in the SPIN group (Fig. 1e). A total of 15 helmets, three per group, were purchased from retailers in size medium for testing at the HIT facility. All helmets represented in-mold constructions, whereby a thin polycarbonate (PC) shell is molded onto an EPS foam core. To characterize key design components of each helmet group, their weight, EPS density and thickness, and PC shell thickness were measured at the frontal helmet aspect that corresponded with the impact location.

### Test Setup

**Figure 2:**
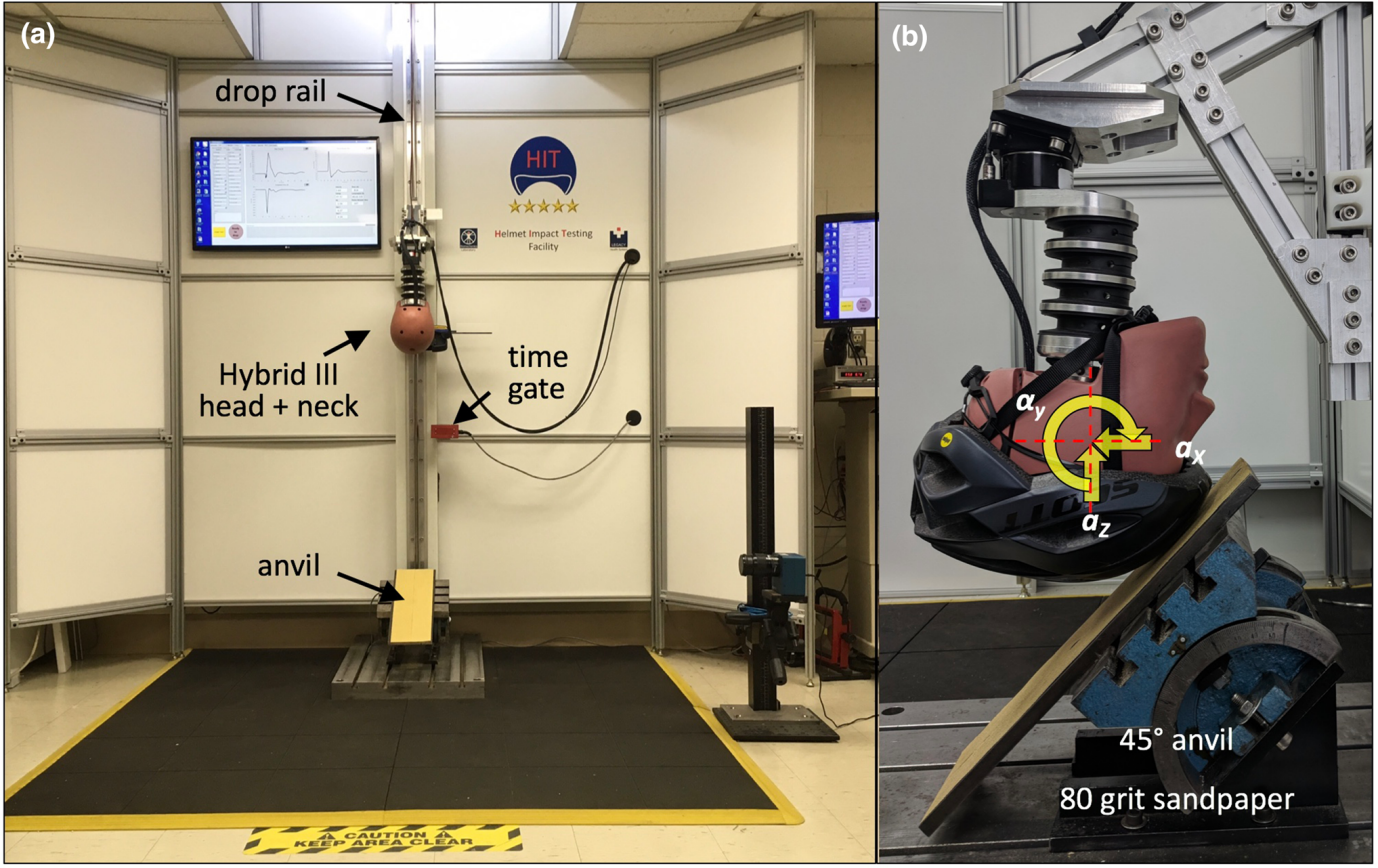
(a) Helmet Impact Testing facility for vertical drop of a Hybrid III head and neck assembly onto a 45° anvil to simulate oblique impacts. (b) Drop assembly with linear and rotational headform accelerometers to capture headform kinematics in terms of linear acceleration (*a*) and rotational acceleration (*α*).

Helmet testing was conducted with a Hybrid III 50th percentile male anthropomorphic head^33^ and neck surrogate^4^,^57^ (78051-336, Humanetic Innovative Solutions, Plymouth, MI) that was connected to a vertical drop tower rail (Fig. 2). A 45° anvil was used to induce oblique impacts in response to vertical drops, in line with the impact angle selected by Bland *et al*. in a recent helmet comparison study.^6^ Linear head acceleration was captured with a three-axis linear accelerometer (356B21 ICP Triaxial, PCB Piezotronics, Depew, NY) mounted at the center of gravity of the Hybrid III head. The resultant linear acceleration *a*_r_ was calculated from the three linear acceleration components. Rotational acceleration *α*_*y*_ and rotational velocity *ω*_*y*_ of the headform around the transverse *y*-axis were measured with a rotational accelerometer (#8838, Kistler Instruments Corp., Amherst, NY). Assessment of headform rotation was limited to rotation around the transverse *y*-axis, since impacts were centered on the sagittal midline of the helmet, and since the anvil surface was aligned parallel to the headform transverse axis.^27^ Impact velocity was measured with a time gate (#5012 Velocimeter, Cadex Inc., Quebec, CA).

Three helmets of each group were tested at 6.2 m/s impact speeds, which correlates with the speed specified in the bicycle helmet safety standard §1203 of the US Consumer Product Safety Commission (CPSC) for impact testing on a flat anvil.^14^ It furthermore falls between the speeds of 5.1 and 6.6 m/s used by Bland *et al*. for oblique impact simulations.^7^ The 6.2 m/s impact speed onto a 45° impact anvil generated equal tangential and normal impact velocities of 4.4 m/s, whereby a tangential velocity of 4.4 m/s simulates a realistic bicycling travel velocity of 16 km/h. The weight of the drop assembly was 14.0 kg, resulting in an impact energy of 269 J. This represents a more severe impact condition than in the CPSC impact test standard, in which impacts of a 5 kg drop assembly at 4.8 and 6.2 m/s result in impact energies of 58 and 96 J, respectively.

Helmets were only tested under ambient conditions, and no other preconditioning environments were considered. The ambient test condition was defined according to the CPSC standard to be within 17 to 27 °C, and 20–80% relative humidity.^14^ Helmets were properly fitted to the headform with their original fit system in accordance with the manufacturers’ fit recommendations. Specifically, helmets were positioned with the front rim approximately 7 cm above the basic transverse plane, which intersects the center of the external ear openings and the lower edge of the eye sockets. Retention straps and fit adjustment dials were securely tightened to firmly retain the helmet position during the free fall. The frontal impact location in the mid-sagittal plane was defined by the vertical alignment of the Hybrid III head and neck surrogate and the 45° impact anvil. Since the silicone skin surrogate of the Hybrid III headform has over twice the surface friction coefficient of the human head,^54^ a nylon stocking was fitted over the Hybrid III headform to reduce surface friction. This approach was adopted from prior studies that utilized the Hybrid III headform in helmeted drop tests.^52^,^55^ All drop tests were performed on a frontal impact location in the mid-sagittal plane to induce headform rotational acceleration around a transverse axis. Before each test, new 80 grit sandpaper was applied to the anvil surface as specified in ECE R-22.05 to simulate road surfaces.^17^

### Data Acquisition and Analysis

Accelerometer data was captured at a sampling rate of 20 kHz in a data acquisition system (PCI-6221, National Instruments, Austin, TX). Accelerations were low-pass filtered at Channel Frequency Class (CFC) 1000.^46^ Rotational velocity *ω*_*y*_ was calculated in LabVIEW software using trapezoidal integration of rotational acceleration data.

The probability of sustaining a concussion was based on an Abbreviated Injury Score (AIS) 2 brain injury, defined as a mild-to-moderate concussion with less than 1 h loss of consciousness. The probability *P*_AIS 2_ of sustaining a type AIS 2 brain injury was calculated according to Eq. (1)^52^:

1 $$ P_{{{\text{AIS}}\;2}} = 1 - e^{{ - \left( {\frac{BrIC}{.567}} \right)^{2.84} }} $$

For Eq. (1), the revised Brain Injury Criterion (*BrIC*) value when using a Hybrid III 50th percentile headform is calculated by Eq. (2), whereby *ω*_*y,*max_ is the peak rotational velocity around a transverse axis, and 56.45 rad/s represents the critical maximal rotational velocity^52^:

2 $$ BrIC = {{\omega_{y,\hbox{max} } } \mathord{\left/ {\vphantom {{\omega_{y,\hbox{max} } } {(56.45\;{\text{rad}}/{\text{s}})}}} \right. \kern-0pt} {(56.45\;{\text{rad}}/{\text{s}})}} $$

Since explicit calculation of head injury criteria from peak loads does not account for loading histories and the temporal and spatial dependency of injury tolerance limits,^57^ acceleration histories were additionally evaluated in the SIMon (Simulated Injury Monitor) finite element model to determine brain strain distributions throughout the impact, and to extract the maximum principal strain *ε*_peak_.^53^ The extensively validated SIMon head model was specifically developed by the U.S. Department of Transportation to advance the interpretation of injury mechanisms based on impact kinematic data collected from anthropomorphic head forms. The head model includes the skull, brain, falx cerebri, bridging veins, and cerebrospinal fluid. While the model renders the head with only 7,852 finite elements, it is computationally efficient, allowing it to generate strain distribution histories for each impact in a timely manner. Accordingly, each of the 15 impact tests were analyzed in SIMon to extract peak brain strain *ε*_peak_.

For statistical analysis, headform kinematics (*a*_r_*, α*_*y*_), the head injury criterion (*P*_AIS 2_), and brain strain (*ε*_peak_) of the 4 helmet groups with rotation-damping systems were compared to CONTROL group results, individually for each outcome parameter. Two-sided Student’s t-tests with Bonferroni correction were used to account for multiple comparisons. A level of *α* = 0.05 was used to detect statistical significance.

## Results

Key design parameters of the 5 helmet groups are summarized in Table 1. EPS density of single-density shells ranged from 74 g/L (ODS) to 100 g/L (SPIN). The total EPS liner thickness ranged from 21 mm (LDL) to 31 mm (ODS). Thickness of the outer PC shell ranged from 0.3 mm (SPIN) to 0.6 mm (ODS). Helmet weight ranged from 208 g (CONTROL) to 522 g (ODS).

**Table 1:** Helmet design parameters: EPS liner thickness at the location of impact.

Helmet technology	EPS density (g/l)	EPS thickness (mm)	PC Shell thickness (mm)	Helmet weight (g)
CONTROL	79 ± 2	32 ± 0.3	0.4 ± 0.02	250 ± 8
MIPS	84 ± 6	32 ± 0.3	0.4 ± 0.03(OS) 0.6 ± 0.02(ML)	263 ± 7
ODS	75 ± 9 (OL) 74 ± 4 (IL)	13 ± 1 (OL) 14 ± 2 (IL)	0.6 ± 0.02 (OS) 0.6 ± 0.03 (IS)	525 ± 4
LDL	51 ± 1 (w) 120 ± 11 (b)^a^	21.2 ± 0.2	0.6 ± 2.9	315 ± 1
SPIN	100 ± 7	30.7 ± 0.2	0.3 ± 0.03	284 ± 6

MIPS helmets had an outer PC shell (OS) and a MIPS liner (ML). ODS helmets had an outer EPS liner (OL) with an outer PC shell (OS), and an inner EPS liner (IL) with inner PC shell (IS). LDL helmets had a dual density EPS liner consisting of white (w) EPS sections that were encapsulated in a black (b) EPS envelope of higher density

^a^The denser black EPS comprised between 54 and 100% of the EPS liner thickness

The average impact speed for all impacts was 6.19 ± 0.03 m/s, and there was no statistically significant difference in the average impact speed between helmet groups.

**Figure 3:**
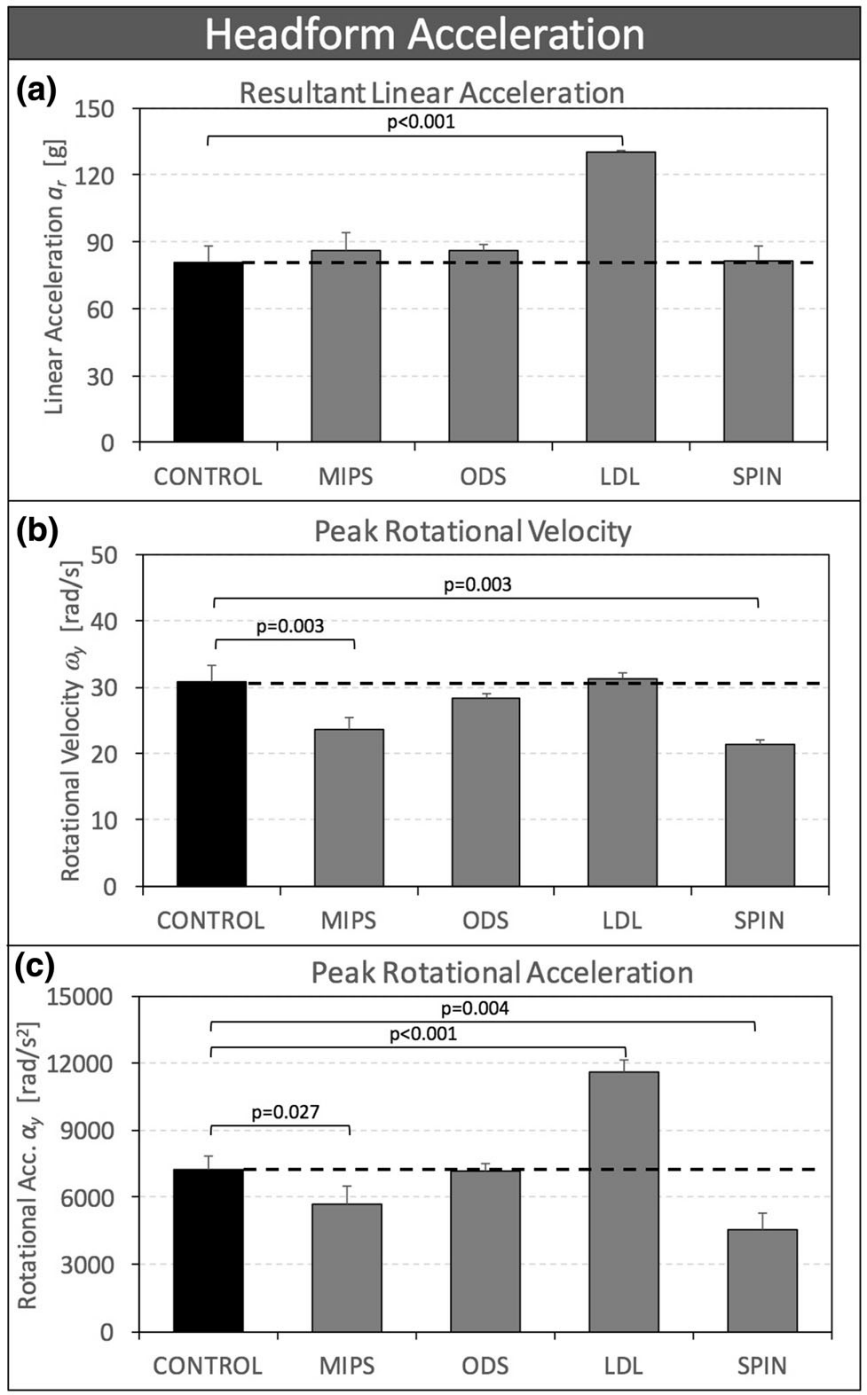
(a) Peak liner acceleration *a*_r_, (b) peak rotational velocity *ω*_r_, and (c) peak rotational acceleration *α*_*y*_ of four helmet designs with rotation-damping systems, shown in comparison to control helmets without a rotation-damping system.

Peak linear acceleration *a*_*r*_ of helmets with an anti-rotation system was not significantly different from that of CONTROL helmets, except for LDL helmets, which exhibited a 62% higher linear acceleration (130 ± 1 g) than control helmets (81 ± 8 g, *p* < 0.001) (Fig. 3a).

Peak rotational velocity *ω*_*y*_ of MIPS and SPIN helmets was significantly reduced by 26% (*p* = 0.003) and 31% (*p* = 0.003), respectively, compared to CONTROL helmets (Fig. 3b). There was no significant difference in *α*_*y*_ between ODS and LDL helmets compared to CONTROL helmets.

Peak rotational acceleration *α*_*y*_ of MIPS and SPIN helmets was significantly reduced by 22% (*p* = 0.027) and 37% (*p* = 0.004), respectively, compared to CONTROL helmets (Fig. 3c). LDL helmets increased *α*_*y*_ by 61% (*p* < 0.001) compared to CONTROL helmets. There was no significant difference in *α*_*y*_ between ODS and CONTROL helmets.

**Figure 4:**
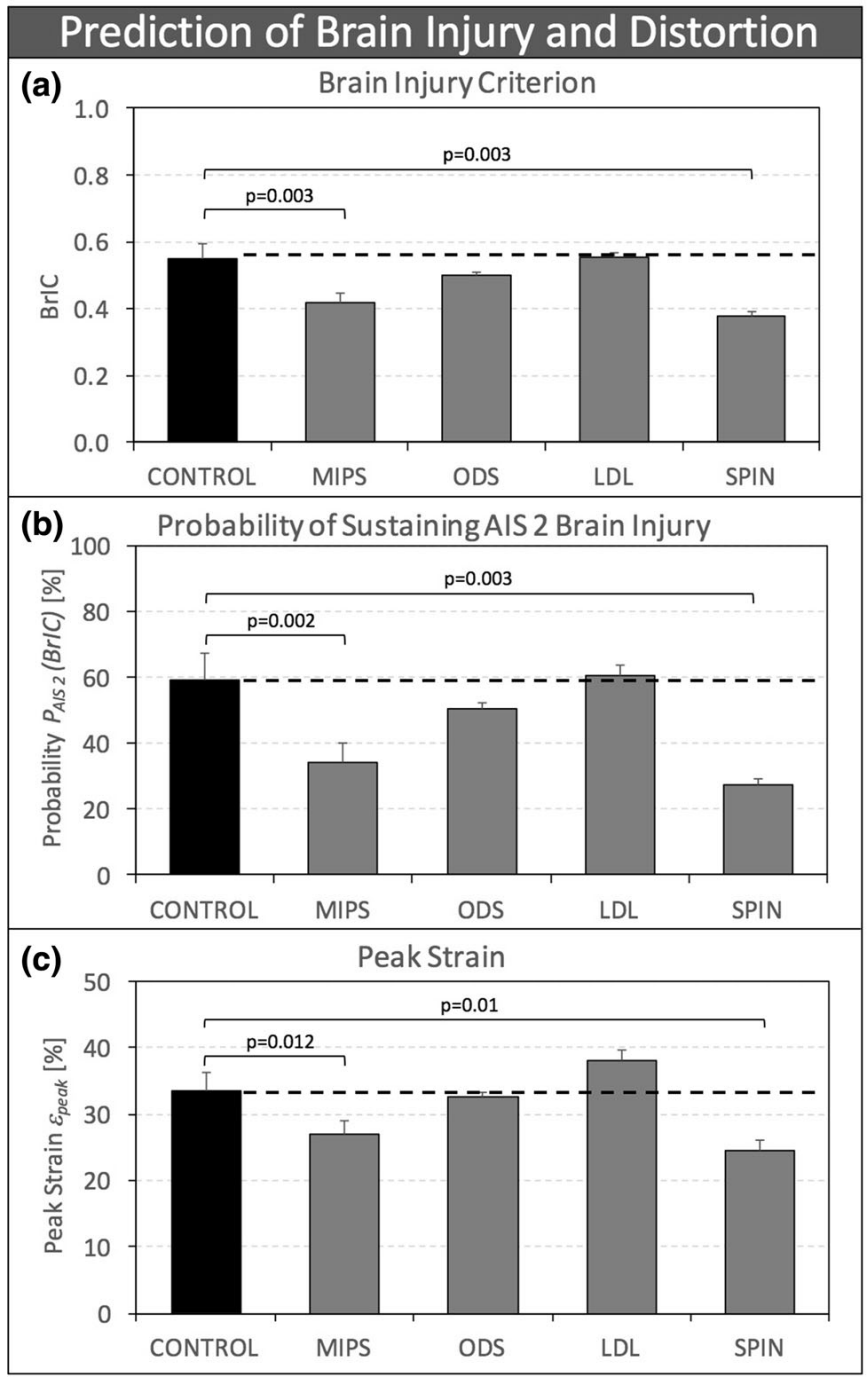
(a) Brain Injury Criterion (BrIC); (b) Predicted probability *P*_AIS 2_ of experiencing AIS 2 brain injury based on BrIC; and (c) peak brain strain *ε*_peak_ computed in the SIMon finite element model for four helmet designs with rotation-damping systems, shown in comparison to control helmets without a rotation-damping system.

BrIC of MIPS and SPIN helmets was significantly reduced by 24% (*p* = 0.003) and 31% (*p* = 0.003), respectively, compared to CONTROL helmets (Fig. 4a). There was no significant difference in *α*_*y*_ between ODS and CONTROL helmets.

The probability *P*_*AIS 2*_ of experiencing AIS 2 brain injury, predicted by BrIC, was significantly reduced by 42% (*p* = 0.002) and 54% (*p* = 0.003) for MIPS and SPIN helmets, respectively, compared to CONTROL helmets (Fig. 4b). There was no significant difference in *P*_*AIS 2*_ between ODS and LDL helmets compared to CONTROL helmets.

Brain strain *ε*_peak_ computed in the SIMon finite element model was significantly reduced by 20% (*p* = 0.012) and 27% (*p* = 0.01) for MIPS and SPIN helmets, respectively, compared to CONTROL helmets (Fig. 4c). There was no significant difference in *ε*_peak_ between ODS and LDL helmets compared to CONTROL helmets.

## Discussion

Oblique impact testing delineated significant performance differences between helmet designs with different rotation-damping systems for the mid-saggital, frontal impact condition tested in this study. Results demonstrated that mitigation of rotational kinematics, concussion risk, and brain strain varied considerably between helmets with different rotation-damping systems. The MIPS and SPIN helmets provided considerable improvements in absorption of rotational kinematics and associated concussion risk compared to control helmets. These highly encouraging results emphasize the need to advance traditional helmet designs towards effective rotation-damping systems that hold a considerable potential for brain injury prevention. Conversely, the ODS and LDL systems did not provide any mitigation of rotational acceleration compared to the CONTROL helmet; in fact, the LDL helmet appeared to increase the angular acceleration and corresponding injury risk compared to the CONTROL helmet. These findings emphasize the need for advanced impact testing of bicycle helmets under real-world oblique impact conditions that capture linear as well as rotational head acceleration and associated brain injury risk. Such testing will be critical to guide developers towards the design of more effective rotation-damping systems, and to educate consumers on helmets that provide the best concussion protection.

Several researchers have previously compared the performance of bicycle helmets in oblique impacts.^8^,^39^,^50^,^51^ At present, the main resource to compare bicycle helmet performance in oblique impact testing is provided by the helmet laboratory at Virginia Tech University. They analyze linear acceleration and rotational velocity of the headform to derive a Summation of Tests for Analysis of Risk (STAR) score and a star rating, ranging from 0 to 5 stars.^5^ As of May 2019, they have tested 64 different bicycle helmets. Since mandatory test standards have not yet embraced oblique impact testing of bicycle helmets, Virginia Tech’s 5-star rating has been a driving force motivating manufacturers to consider rotation-damping systems. In addition, it provides clear consumer guidance by reducing helmet impact performance to a simple 5-star rating. In contrast to consumer guidance, the present study was designed to report helmet performance in greater detail, suitable for researchers and developers to aid in the design and testing of helmets with rotation-damping systems. For this purpose, it also includes measurements of helmet design parameters.

In the present data set, MIPS and SPIN rotation-damping systems demonstrated significant improvements in mitigation of rotational kinematics and consequential brain injury risk and brain strain. These rotation-damping systems were not designed to affect linear acceleration, and the linear acceleration results were remarkably consistent with those of the standard CONTROL helmets. ODS helmets did not significantly differ from CONTROL helmets in any of the four outcome parameters. This may be attributed to the large spacing between elastic dampers that separate the outer and inner EPS liners of ODS helmets, with no elastic damper being present at the impact location. This allowed direct contact between the inner and outer EPS liners, yielding an impact performance similar to the singular EPS shell in the CONTROL group. LDL helmets were the only helmets that exhibited a significantly higher linear acceleration than CONTROL helmets. This may be attributed to the dual-density EPS liner of LDL helmets, which was thinner than that of CONTROL helmets, whereby 54–100% of the LDL liner thickness comprised black foam that was approximately 60% denser than the EPS liner of CONTROL helmets. This thinner and stiffer EPS liner may absorb less impact energy, and in turn may have contributed to the significantly increased rotational acceleration. Compared to CONTROL helmets, LDL helmets had a significantly higher rotational acceleration, but a similar peak rotational velocity. This may explain why the predicted brain injury risk of LDL helmets was comparable to CONTROL helmets, since BrIC and *P*_AIS 2_(BrIC) are based on peak rotational velocity.

While these results demonstrate that the test method can delineate performance differences between rotation-damping systems, they are limited to a specific impact location, impact angle, and impact velocity. Therefore, they cannot be extrapolated or generalized outside this specific impact scenario. For example, testing of ODS helmets at an impact site coinciding with an impact damper element would likely have yielded different results. However, the present results are relevant, since the helmet front is a commonly impacted region,^11^ which is involved in over 50% of bicycle helmet impacts.^9^ A mid-sagittal impact location was chosen to simplify the impact kinematics, and to match the impact scenarios in previously published studies.^1^,^21^,^27^,^30^,^39^ Nevertheless, future testing will be expanded to include a range of impact locations to capture a more comprehensive assessment of helmet performance.

Peak linear acceleration of helmets in the present study ranged from 81-130 g. This linear acceleration is far below the 300 g linear acceleration threshold mandated by the CPSC safety standard, which was developed to prevent skull fractures.^14^ A rotational head acceleration of 5.9 krad/s^2^ has been correlated to a 50% probability of sustaining a concussion.^58^ Rotational acceleration results in the present study ranged from 4552 to 11,632 rad/s^2^ and correlated with a rotational acceleration range of 3348–11,682 rad/s^2^ reported by Bland *et al*. in a recent helmet comparison study.^7^ They tested 10 different helmet models in oblique impacts onto a 30° anvil at impact speeds of 5.1 and 6.6 m/s. While they employed the same Hybrid III neck as the present study, they used a different headform type and orientation. In the study by Bland *et al*., two of the 10 helmet models contained MIPS slip liners.^7^ These two MIPS helmet models resulted on average in a peak rotational acceleration of 6.0 krad/s^2^, while the eight helmet models without MIPS liner resulted on average in a peak rotational acceleration of 5.3 krad/s^2^. This suggests that the performance of slip liners may depend on many factors, ranging from helmet design to test methodology, such as impact parameters, choice of headform, and headform constraints. Therefore, performance improvements observed for MIPS helmets in the present study may not be extrapolated to other helmets with MIPS slip liner technology.

Results of this study are limited to a specific test configuration and may not be extrapolated outside these test parameters. Results are specific for a Hybrid III 50th percentile male anthropomorphic head, which was chosen because it is the most widely used human head surrogate employed for impact testing.^4^ It provides an elastic skin envelope, and its inertial properties are considerably more biofidelic than those of ISO headforms specified in the CPSC safety standard.^57^ While the headform of the National Operating Committee on Standards for Athletic Equipment (NOCSAE) is considered to have the most biofidelic headform shape, integration of a neck and instrumentation is more difficult compared to a Hybrid III head.^12^

While there is precedent for impact testing using an unconstrained headform without a neck surrogate,^21^,^33^,^39^,^40^ the present study simulated quasi-physiologic head constraints with a Hybrid III neck.^7^ The Hybrid III neck was validated for flexion and extension, but has been shown to be overly stiff in lateral bending and axial compression.^48^ The Hybrid III head and neck combination has been used in a range of helmet impact studies^4^,^7^,^27^,^36^,^44^ and has been proposed for advanced testing of bicycle helmets.^57^ The experimental design was limited to a specific impact angle, location, and speed. The 45° impact angle correlated with the middle of the 30°–60° range determined from reconstruction of real-world bicycle accidents.^1^,^9^,^10^ The 6.2 m/s impact speed aligned with the impact speed specified in the CPSC standard for impact testing on flat anvils (6.2 m/s).^14^ However, this impact speed is likely conservative, considering average impact speed of the helmeted head with a car or the road has been reported to be between 6.4 and 6.9 m/s.^9^,^10^ Most importantly, the impact test of this study was designed to assess mitigation of rotational headform kinematic caused by the tangential velocity component during an oblique impact. However, the head can also exhibit rotational forces from a normal, non-oblique impact to the side of the helmet, which causes the head to rotate around the lower neck. Moreover, brain injury can also occur from inertial loading in absence of a direct impact to the head. In these cases, rotation-damping systems may have less or no effect on mitigation of rotational headform kinematics and associated TBI risk.

In addition to limitations due to simplified simulation of real-world impacts under reproducible laboratory conditions, further limitations must be considered when predicting brain injury risk from impact kinematics data. Prediction of brain injury risk from BrIC and axonal strain depends on the accuracy of injury risk functions that have been reconstructed from a limited number of real-world injury data to estimate brain tolerance limits. Brain strain *ε*_peak_ computed in the SIMon finite element model ranged from 25% (SPIN) to 38% (LDL). For correlation, thresholds for reversible and irreversible axonal injury range from 7 to 15% axonal strain,^24^ and 14–34% axonal strain,^3^ respectively. A 21–26% maximum principal strain has been correlated with a 50% concussion risk.^32^ Moreover, 15% axonal strain has been associated with a 50% risk of AIS 2 Diffuse Axonal Injury (DAI).^47^ Estimating the probability *P*_*AIS2*_ of sustaining a concussion based on BrIC values strongly depends on the injury risk curve. The present study employed the injury risk curve of Takhounts *et al.*^52^ Laituri *et al.* proposed a revised injury risk curve for BrIC values,^34^ which leads to considerably lower injury risk probabilities, whereby the results of the present study would fall within the toe region of their revised injury risk curve. While the uncertainty in defining injury risk curves necessarily limits the accuracy in predicting an absolute probability of brain injury, assessment of brain strain and predictors of brain injury risk can be useful for relative comparisons between helmet technologies.

In conclusion, results demonstrated that some rotation-damping systems of advanced bicycle helmets can significantly reduce rotational head acceleration and associated TBI risk. However, our results also demonstrated significant differences in effectiveness among rotation-damping systems, emphasizing the need for advanced impact testing of bicycle helmets under real-world oblique impacts.
